# A Rare Case of Sodium-Glucose Cotransporter-2 Inhibitor-Induced Acute Pancreatitis

**DOI:** 10.7759/cureus.49369

**Published:** 2023-11-24

**Authors:** Rakahn Haddadin, Roger F Tonna, Humzah Iqbal, Jordan Valenta, Homayon Iraninezhad

**Affiliations:** 1 Internal Medicine, MountainView Hospital, Las Vegas, USA; 2 Internal Medicine, University of California San Francisco, Fresno, Fresno, USA; 3 Gastroenterology, MountainView Hospital, Las Vegas, USA

**Keywords:** gastrointestinal, medication side-effects, sodium-glucose cotransporter-2 (sglt-2) inhibitors, diabetes type 2, medication-induced pancreatitis

## Abstract

Acute pancreatitis is an acute inflammatory process of the pancreas that requires hospital admission and treatment. There are many causes of pancreatitis, the most common being gallstone and alcohol-induced; other reasons include metabolic, infectious, and medication-induced. A new medication that has come to the market is empagliflozin, which is a sodium-glucose cotransporter-2 inhibitor that is common in managing type 2 diabetes mellitus and congestive heart failure. Although generally considered safe and effective, rare adverse effects have been reported. In this case, we present a 67-year-old female patient who presented with severe acute pancreatitis after two weeks of starting empagliflozin to treat her type 2 diabetes. This case report highlights the importance of considering rare adverse events associated with empagliflozin and the need for close monitoring of patients receiving this medication.

## Introduction

Acute pancreatitis is an inflammatory reaction within the pancreatic tissue that accounts for a significant amount of morbidity and healthcare expenditure [1]. Common etiologies include gallstones, alcohol, trauma, steroids, autoimmune disease, hypercalcemia, hypertriglyceridemia, endoscopic retrograde cholangiopancreatography (ERCP), and drugs [2]. Medication-induced pancreatitis is a common precipitant, but identifying and showing an association can be complex [2]. Empagliflozin is a sodium-glucose cotransporter-2 (SGLT-2) inhibitor approved for treating type 2 diabetes mellitus (T2DM). SGLT-2 inhibitors reduce blood glucose levels by inhibiting renal glucose reabsorption [3]. Empagliflozin has been generally well-tolerated, with common adverse effects such as urinary tract infections and genital mycotic infections [4]. However, reports of empagliflozin-induced pancreatitis are exceedingly rare [5].

## Case presentation

We present a 67-year-old female with a past medical history of T2DM, hypertension, dyslipidemia, and atrial fibrillation on apixaban, who was admitted to the hospital for intractable abdominal pain, shortness of breath, and chest pain. The patient denied any alcohol or drug use or any family history of pancreatitis and stated she was retired but previously worked an office job. The patient's initial labs on presentation were aspartate aminotransferase (AST) of 20 U/L, alanine transaminase (ALT) of 19 U/L, alkaline phosphatase (ALP) of 86 U/L, and lipase of 26 U/L. The patient was previously admitted into the hospital the week prior for similar symptoms and was found to have AST of 19 U/L, ALT of 17 U/L, ALP of 53 U/L, total bilirubin of 0.9 mg/dL, lipase of 254 U/L along with abdominal and back pain, and CT findings concerning for pancreatitis.

**Figure 1:**
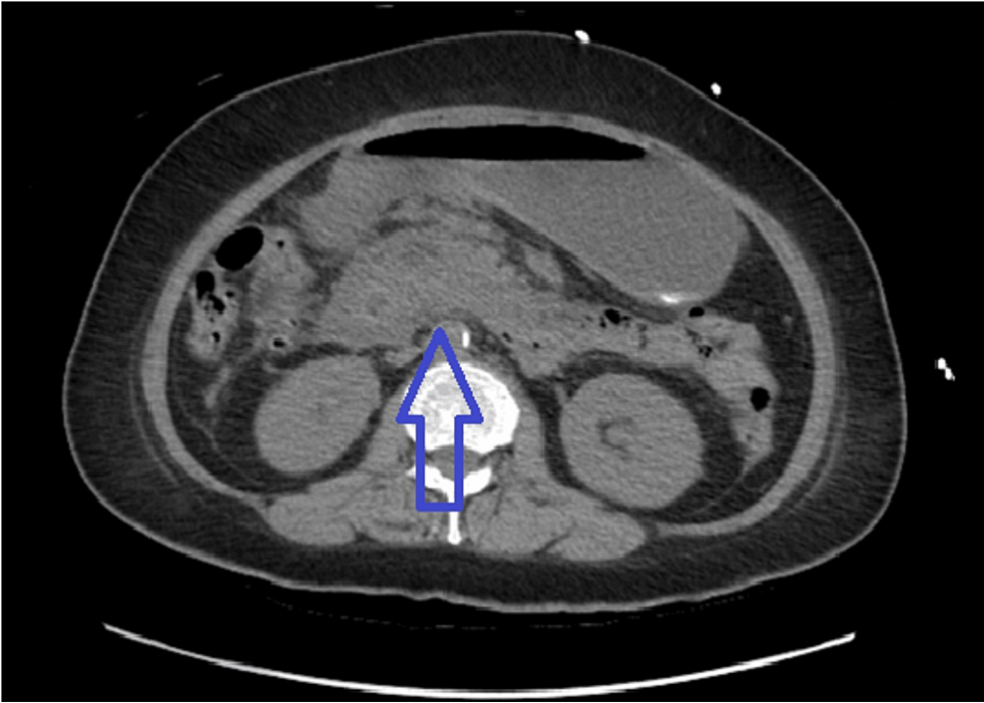
Increased edematous changes at the pancreas consistent with acute pancreatitis.

The patient was treated with IV fluids and morphine for pain control and was discharged to follow up with GI outpatient and return to the hospital if symptoms persisted. On this admission, the patient described the pain as mid-epigastric with radiation to the back that worsens with positional movement. The patient had multiple episodes of nausea and vomiting that worsened with drinking and eating. The patient's pain was relieved with complete restriction of diet and liquids and intravenous morphine for pain medication (IV). The patient denied any alcohol use, previous history of gallstones, recent trauma, or autoimmune disease history. When reviewing the patient's home medication, it was noted for atenolol 50 mg, apixaban 5 mg, and most recently, empagliflozin 25 mg daily, which the patient started two weeks before initial hospital presentation and symptom onset. The patient's vitals were stable on arrival except for mild tachycardia of 101 beats per minute. Physical examination was significant for severe tenderness to palpitation in the midepigastric region. A computed tomography (CT) scan of the abdomen in the ED was noted for increased edematous changes in the pancreas consistent with acute pancreatitis (Figure 1).

**Figure 2:**
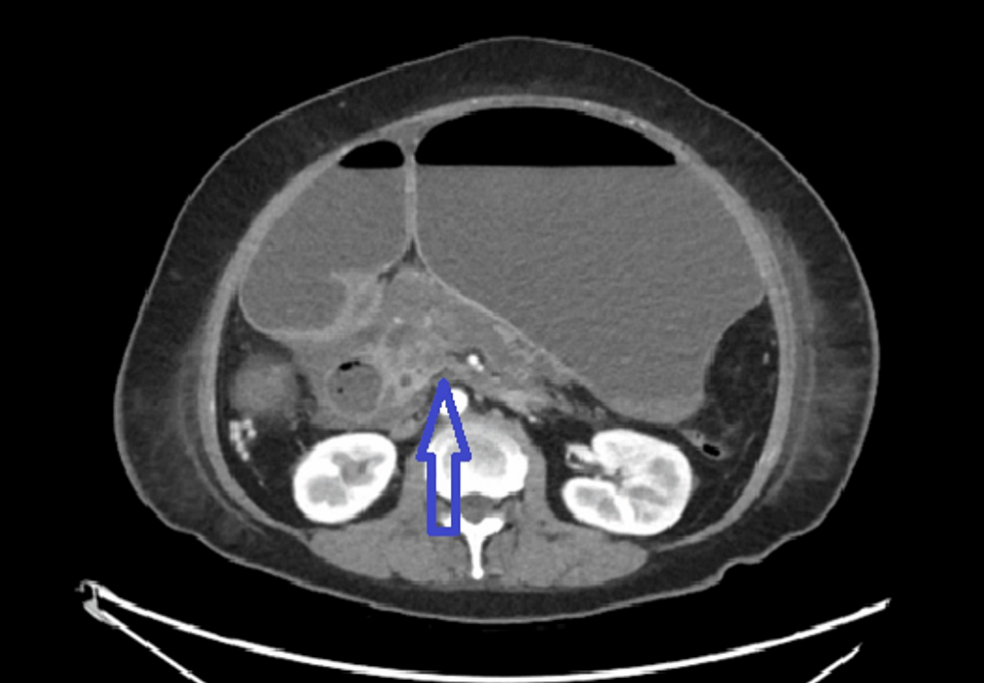
Slightly heterogeneous attenuation in the region of the pancreatic head and uncinate process associated with moderate peripancreatic fat stranding and fluid, most in keeping with pancreatitis. The small areas of diminished attenuation in the pancreatic head and uncinate process reflect changes of pancreatic necrosis. There is no discrete fluid collection to suggest pancreatic pseudocyst or abscess at this time.

The patient was admitted with a diagnosis of acute pancreatitis and was started on conservative treatment with nothing by mouth, IV fluid, antiemetics, and a pain regimen that consisted of IV morphine. Additional labs showed a fasting lipid panel of triglyceride levels within normal limits and a calcium level of 8.1. Glucose level was 188 mg/dL on admission with high ketone levels in urine and metabolic acidosis. The patient did have euglycemic diabetic ketoacidosis (euDKA), which was corrected. The patient still had abdominal pain, nausea, and vomiting. Upon the patient's pain worsening, a repeat CT abdomen pelvis with contrast showed possible pancreatic necrosis in the pancreatic head and uncinate process along with moderate peripancreatic fat stranding and fluid in the region of the head and uncinate process (Figure 2). Additionally, we calculated a bedside index of severity of acute pancreatitis (BiSAP) score which came out to be 3 points; 1 point for blood urea nitrogen (BUN) >25, altered mental status (AMS) > systemic inflammatory response syndrome (SIRS). No alcohol level was ordered. A magnetic resonance cholangiopancreatography (MRCP) done during hospitalization showed acute pancreatitis with a surrounding phlegmonous process with proteinaceous/hemorrhagic products. There were no filling defects within the biliary system identified and no intra or extrahepatic biliary dilatation.

Empagliflozin was discontinued upon diagnosis, and the patient was managed with supportive care, including being placed on nothing per mouth and intravenous fluids. For glucose control in the hospital, the patient was started on a mild insulin sliding scale, which she tolerated well during her time. Upon discharge, the patient was recommended to discontinue the empagliflozin and continue her atenolol and apixaban.

## Discussion

Drug-induced pancreatitis is a rare but potentially severe adverse event associated with several medications, including certain antidiabetic drugs [6]. Some common medications that are related to drug-induced pancreatitis include angiotensin-converting enzyme inhibitors, statins, hormone replacement therapy, diuretics, and other anti-glycemic agents such as dipeptidyl peptidase (DPP4) inhibitors and glucagon-like peptide-1 (GLP-1) mimetics [7].

The mechanism of action of SGLT-2 inhibitors involves altering the kidney’s reabsorption of glucose. Plasma glucose is freely filtered by the glomerulus and reabsorbed in the proximal renal tubule, with a small amount excreted directly in the urine. It is reported that ninety percent of renal glucose reabsorption occurs in the proximal tubule due to SGLT-2 [7]. Very few cases of SGLT-2 inhibitor-induced pancreatitis have been reported in the literature [8].

While the exact mechanism of empagliflozin-induced pancreatitis remains unclear, it is essential to consider this possibility, particularly in patients with new-onset abdominal symptoms after initiating the drug. One possible explanation for this adverse effect involves the fact that SGLT-2 inhibitors work by inhibiting the kidney's capacity to reabsorb glucose, thus decreasing plasma glucose levels [9]. These alterations in glucose metabolism caused by SGLT-2 inhibitors may potentially lead to inflammation and pancreatic tissue injury. The proposed mechanisms of how SGLT-2 inhibitors cause pancreatitis include direct toxicity to pancreatic cells, immune-mediated response, or idiosyncratic reactions [10].

In our case, the patient's only new medication was empagliflozin for better glycemic control. The patient's other medications were atenolol and apixaban, which were chronically taken, and upon further literature review, there was no evidence for either to cause pancreatitis [11]. It is possible to perform a drug re-challenge, which is defined as the re-administration of a suspected drug that had been previously withdrawn; this would further strengthen our case to prove an association between SGLT-2 inhibitors and pancreatitis [12].

The temporal relationship between empagliflozin initiation and the onset of pancreatitis strongly suggests a potential causal association. This makes the current case presentation unique and essential for furthering medical knowledge on the side effects of SGLT-2 inhibitors. Another strong indication that the patient suffered from medication-induced pancreatitis was the findings of normal liver function tests. CT and MRCP findings too did not note, or suggest, any obstructive processes occurring that could lead to pancreatitis. In addition, the patient had no other risk factors for pancreatitis such as alcohol use or trauma, making drug-induced pancreatitis the most probable explanation.

## Conclusions

Empagliflozin is generally considered safe and effective for glycemic control in patients with T2DM. With the increasing popularity of the SGLT-2 inhibitor class, clinicians should be aware of the rare adverse events such as pancreatitis associated with this medication. Monitoring for signs and symptoms of pancreatitis, especially in patients with new-onset abdominal pain or gastrointestinal symptoms after starting empagliflozin, is crucial. Prompt recognition and appropriate management are essential to ensure the best outcomes for affected individuals. With the patient having started the empagliflozin two weeks prior to symptom onset, it leads to a high likelihood that it has caused the acute pancreatitis in this patient. Further research in the form of retrospective studies on the association of SGLT-2 inhibitor-induced acute pancreatitis is indicated to examine the direct relationship between all SGLT-2 inhibitors and acute pancreatitis. It will be vital to see if all SGLT-2 inhibitors carry the same amount of risk for acute pancreatitis or not.
